# Advanced Parkinson’s disease treatment patterns in Italy: results from a multicenter observational study

**DOI:** 10.1080/07853890.2026.2628353

**Published:** 2026-03-04

**Authors:** Fabrizio Stocchi, Paolo Barone, Roberto Ceravolo, Maria Francesca De Pandis, Leonardo Lopiano, Nicola Modugno, Alessandro Padovani, Manuela Pilleri, Alessandro Tessitore, Mario Zappia

**Affiliations:** a Department of Neurology, University San Raffaele Roma and IRCCS San Raffaele, Rome, Italy; b Department of Medicine, Surgery and Dentistry "Scuola Medica Salernitana," Neuroscience Section, University of Salerno, Baronissi, Italy; c Neurodegenerative Disease Center, Department of Clinical and Experimental Medicine, University of Pisa, Pisa, Italy; d Department of Human Sciences and Promotion of Quality of Life, San Raffaele University, Roma, Italy; e IRCCS San Raffaele Roma, Cassino, Italy; f Department of Neuroscience Rita Levi-Montalcini, University of Turin; AOU Città della Salute e della Scienza, Turin, Italy; g I.R.C.C.S. Neuromed, Pozzilli, Isernia, Italy; h Neurology Unit, Department of Clinical and Experimental Sciences, University of Brescia, Brescia, Italy; i UO Neurologia Casa di Cura Villa Margherita, Arcugnano Vicenza, Italy and Centro Parkinson e Parkinsonismi ASST Gaetano Pini CTO, Milano, Italy; j Department of Advanced Medical and Surgical Sciences, University of Campania “L. Vanvitelli”, Naples, Italy; k Department “G.F. Ingrassia”, University of Catania, Catania, Italy

**Keywords:** COMT inhibitor, dyskinesia, fluctuation, levodopa, MAOB inhibitor, opicapone, Parkinson disease, safinamide, wearing-off

## Abstract

**Background and objectives:**

Substitution therapy with oral levodopa is the primary treatment of Parkinson’s disease (PD). However, long-term levodopa use is associated with fluctuations in response and dyskinesia. These complications severely affect the patient quality of life. Fluctuation management in long-standing PD is poorly documented. The Parkinson’s Disease Fluctuations treatment Pathway (PD-FPA) study was an Italian multicenter, observational study designed to describe how fluctuations are treated in patients with advanced disease.

**Patients and methods:**

Between July 2018 and December 2020, ten centres enrolled consecutive patients aged ≥18 years who had been diagnosed with PD 10–15 years before enrollment and had been experiencing fluctuations for at least 2 years before enrollment. Data on patient characteristics, PD stage, fluctuations, and treatments were collected at enrollment (T0) and prospectively at 6 months (T1) and 12 months (T2). Data were also collected retrospectively, at 1 and 2 years before T0.

**Results:**

At T0, patients (*n* = 296, 60.1% male, mean age 68 years) had Hoehn and Yahr disease stage 2–3 and 47% had comorbidities (29.8% cardiovascular disease). PD stage and other PD assessment scores were overall stable during the entire 3-year observation period. Over 3 years, the use of dopamine agonists progressively decreased (51% of patients at T2), the use of monoamine oxidase-B inhibitors was stable (63%), while the use of catechol-O-methyltransferase inhibitors progressively increased (42%). Safinamide and opicapone showed the biggest increase in use over the 3-year observation period. Treatment changes were mostly prompted by fluctuations and were reported in about 50% of patients at T0 and 30% at T1 and T2.

**Conclusion:**

Maintenance of stable disease in patients with long-standing PD and fluctuations is feasible with non-invasive treatments. Accurate treatment adjustments and individualized strategies with new-generation add-on drugs may be of key importance.

## KEY MESSAGES

- The management of fluctuations in long-standing PD is poorly documented.
- The Parkinson’s Disease Fluctuations treatment Pathway (PD-FPA) study was an Italian multicenter, observational study designed to describe how fluctuations are treated in patients with advanced disease.
- Our results suggest that oral medications can ensure the maintenance of an acceptable quality of life even in patients with advanced disease, provided patients are followed-up regularly and therapy adjustments are made as necessary.

## Introduction

Since its first use in the early 1960s for the treatment of Parkinson’s disease (PD), dopamine substitution therapy with oral levodopa continues to be the most effective primary treatment of this condition [1–3].

However, long-term use of levodopa is associated with motor fluctuations and dyskinesias [4–6]. The prevalence of wearing-off, a common type of fluctuation, increases with disease duration [7]. Motor fluctuations have a detrimental effect on the patient quality of life [8–10].

Improving wearing-off symptoms without increasing the frequency of levodopa-induced dyskinesias can be challenging [4]. Current strategies for reducing fluctuations include increasing the dose of levodopa, shortening the intervals between levodopa dosing, and introduction of dopamine agonists (DA), monoamine oxidase-B inhibitors (MAOBi) and inhibitors of the enzyme catechol-O-methyltransferase (COMT, COMTi) [1,2,11]. For patients with persistent fluctuations, advanced therapies can be considered, including deep brain stimulation (DBS), levodopa-carbidopa intestinal gel (LCIG) infusions, and continuous infusions of apomorphine [12].

There is limited published evidence about the management of PD patients with long-standing PD and motor fluctuations [13–15]. In order to fill this gap, the multicenter, observational Parkinson’s Disease Fluctuations treatment Pathway (PD-FPA) study was designed to describe how fluctuations are treated in Italy, with a focus on patients with advanced PD [16]. Results of the interim analysis of this study have suggested that accurate levodopa dosing and adjunctive medications can ensure prolonged disease stability and quality of life maintenance [16]. Here we report the results of the final analysis of the PD-FPA study.

## Materials and methods

### Study design and patients

**Figure 1.**
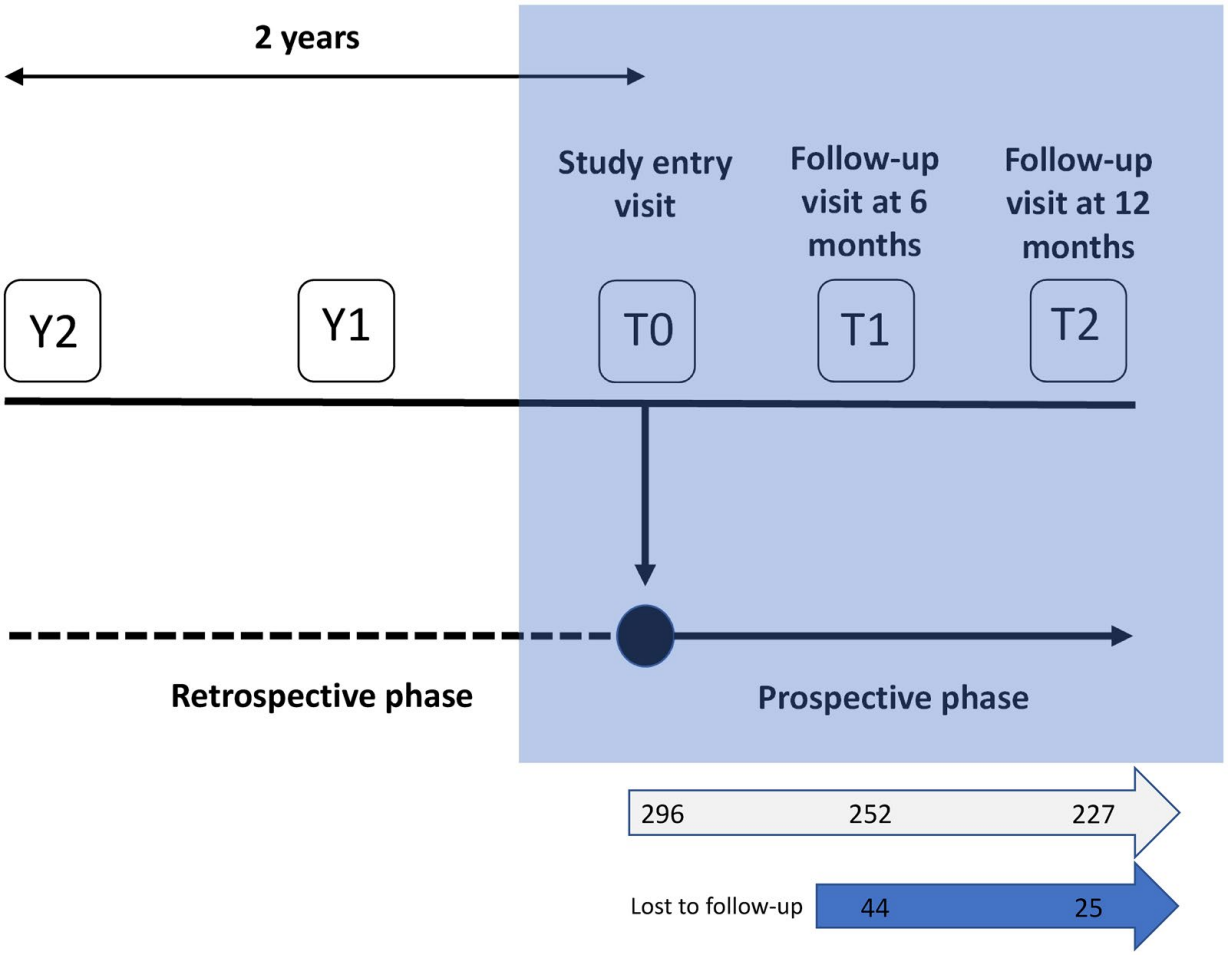
Study design and patient disposition (this report is focused on the prospective phase of the study). T0: study entry; T1: approximately 6 months since study entry; T2: approximately 12 months since study entry; Y1: approximately 1 year before study entry; Y2: approximately 2 years before study entry.

The PD-FPA study was a multicenter, observational study that included both a retrospective and a prospective phase (Figure 1). The results of a planned interim analysis of the PD-FPA, focused on retrospective data, have been published previously [16]. Herein, we present the results of the prospective phase of the study.

The goal of the PD-FPA study was to describe real-life management of motor fluctuations in patients with PD, in Italy [16]. Ten hospitals (four in Southern Italy, two in Central Italy, and four in Northern Italy) were involved in the study, all of which are recognized as PD reference centers and are located across the entire national territory. Between July 2018 and December 2020, each center enrolled consecutive patients aged ≥ 18 years who had been diagnosed with PD 10–15 years before enrollment and had been experiencing fluctuations in their response to oral levodopa for at least 2 years before enrollment. Patients were ineligible for enrollment if they had undergone neurosurgery (i.e. DBS). Patients who withdrew consent or were lost to follow-up were not included in the final analysis. Prospective data collection ended in December 2021. The study was conducted according to the Declaration of Helsinki principles and was approved by the ethics committee of the IRCCS San Raffaele Pisana, Rome, Italy (05/2018). Written informed consent was obtained from all patients at enrollment.

### Study objectives

The primary objective of the PD-FPA study was to describe the characteristics of patients with long-standing PD and motor fluctuations, and to provide an overview of treatments used in clinical practice for this subgroup of PD patients.

### Variables analyzed

The following variables were collected at study entry (T0; Figure 1) and retrospectively: demographic characteristics, medical history, PD stage, characteristics of motor fluctuations, symptoms (motor and non-motor), PD treatment, and comorbidities [16]. The retrospective review of patient charts considered two different time points: Y2, approximately 2 years before T0, and Y1, approximately 1 year before T0. During the prospective phase of the study, patients were monitored for 1 year, with follow-up visits scheduled at approximately 6 months (T1) and 12 months (T2) from the initial visit at T0 (Figure 1). A period of 6 months between T1 and T2 was chosen because it corresponds to the typical interval between visits in the management of patients with advanced PD in clinical practice. Due to the COVID-19 pandemic, the evaluations at T1 and T2 were performed remotely *via* telemedicine, except for motor assessment, which required a face-to-face interaction between physician and patient. All collected data were recorded in case report forms specifically designed for the study. Table S1 (Supplementary materials) provides an overview of all data collected from Y2 to T2.

### Assessments

Clinical assessments were performed at T0 and during the two follow-up visits at T1 and T2 (Figure 1) [16]. Disease stage was established based on physician’s judgement and PD assessment; staging using the Hoehn and Yahr scale was performed within the motor examination of the Unified Parkinson’s Disease Rating Scale revised by the Movement Disorder Society (MDS-UPDRS). The Hoehn and Yahr stages are defined as follows: 0, asymptomatic; 1, unilateral involvement with minimal or no functional impairment; 2, bilateral involvement without impairment of balance; 3, mild to moderate disability with some postural unsteadiness; 4, fully developed disease with severe disability, the patient is still able to stand and walk; and 5, the patient is confined to bed or wheelchair unless aided [17,18].

Fluctuations were evaluated using the Italian version of the self-administered 19-item Wearing-Off Questionnaire (WOQ-19) [19,20]. The WOQ-19 has questions about the presence of 19 motor and non-motor PD symptoms and whether these symptoms improve following medication.

Motor and non-motor symptoms of PD were evaluated using the MDS-UPDRS [18], the Non-Motor Symptom Scale (NMSS) [21,22], and the Italian version of the self-administered Parkinson’s Disease Questionnaire (PDQ-39) [23–25]. The MDS-UPDRS comprises four sections: Part I (non-motor experiences of daily living), Part II (motor experiences of daily living), Part III (motor examination), and Part IV (motor complications) [18]. Part I has two sections: IA, completed by the clinician with input from patients and caregivers, and IB, a patient self-report. Part II, like part IB, is self-administered. The 30-item NMSS questionnaire comprises nine dimensions: cardiovascular, sleep/fatigue, mood/cognition, perceptual problems, attention/memory, gastrointestinal, urinary, sexual function, and miscellaneous items [21]. The self-administered PDQ-39 questionnaire evaluates the impact of PD on quality of life *via* 39 items covering eight dimensions: mobility, activities of daily living, emotional well-being, stigma, social support, cognition, communication, and bodily discomfort [25]. The scale of each dimension ranges from 0 (no impact of symptoms) to 100 (maximum impact).

### Statistical analysis

Data from all study time points were analyzed using descriptive statistics [16]. Categorical variables are presented as frequency and percentages, while continuous variables are summarized using mean/median and standard deviation (SD) values. The statistical software SAS version 9.4 (SAS Institute Inc., Cary, NC, USA) was used for analyses.

## Results

### Patient demographic and clinical characteristics

At study entry, 296 patients were enrolled; the demographic and clinical characteristics of the study population are summarized in Table 1. The patient population was predominantly male (60%) and had a mean age of 68 years; they had been diagnosed with PD at a mean age of 56 years and had experienced the first fluctuations at a mean age of 62.6 years. At T0, most patients (>80%) had a Hoehn and Yahr disease stage in ON between 2 and 3 (i.e. they were physically independent with mild-to-moderate bilateral involvement and some postural instability). Comorbidities were present in 47% of patients (46% at T1 and 50% at T2); the most prevalent comorbidity was hypertension (21%), followed by anxiety/depression (10%) and heart disease (9%). This pattern of comorbidities was maintained over the 1-year period of prospective observation. Of the 296 patients enrolled in the study, 227 completed follow-up; 44 (15%) and 25 (9%) were lost to the follow-up visits at T1 and T2, respectively (Figure 1).

**Table 1.** Patient demographic and clinical characteristics at T0 (*N* = 296).

Characteristics	Patients (*N* = 296)	
	*n/N* (%)	Mean (±SD)
Sex		
Male	178/296 (60.1)	–
Female	118/296 (39.9)	–
Age, years		
At T0	–	68.0 (9.7)
At diagnosis	–	56.1 (9.0)
At first fluctuation	–	62.6 (10.6)
Oral levodopa use		
Yes	294/296 (99.3)	–
Total daily dose, mg	–	662.2 (271.5)
Number of daily administrations	–	5.6 (1.9)
Time since last dose, min	–	130
LCIG use	13/296 (4.4)	
Hoehn and Yahr stage^a^		
0	1/296 (0.3)	–
1	8/296 (2.7)	–
2	127/296 (42.9)	–
3	120/296 (40.5)	–
4	33/296 (11.1)	–
5	6/296 (2.0)	–
Motor examination score^b^ MDS-UPDRS, Part III		
All	–	37.6 (16.2)
Functional state ON	–	34.8 (14.9)
Functional state OFF	–	48.1 (16.2)
NMSS	–	59.1 (43.2)
PDQ-39	–	35.0 (14.7)
With comorbidities	139/296 (47.0)	–
Comorbidities affecting ≥5% of patients		
Hypertension	63/296 (21.3)	–
Anxiety/depression	29/296 (9.8)	–
Heart disease^c^	25/296 (8.5)	–
Benign prostatic hyperplasia	20/296 (6.8)	–
Dyslipidemia/hypercholesterolemia	19/296 (6.5)	–
Gastritis/gastroesophageal reflux	19/296 (6.5)	–
Diabetes	18/296 (6.0)	–
Osteoporosis	15/296 (5.0)	–

^a^ PD stages according to Hoehn and Yahr (Part III of MDS-UPDRS): 0, Asymptomatic; 1, Unilateral involvement only; 2, Bilateral involvement without impairment of balance; 3, Mild to moderate involvement; some postural instability but physically independent; needs assistance to recover from pull test; 4, Severe disability; still able to walk or stand unassisted; 5, Wheelchair bound or bedridden unless aided. [MDS-UPDRS].

^b^ Score according to MDS-UPDRS, Part III (range 0–108).

^c^ Angina, arrythmia, hypertensive heart disease, ischemic heart disease, atrial fibrillation, heart failure, valvular heart disease.

LCIG: levodopa-carbidopa intestinal gel; MDS-UPDRS: Unified Parkinson’s Disease Rating Scale by the Movement Disorder Society; NMSS: Non-Motor Symptom Scale; SD: standard deviation; PDQ-39: Parkinson’s Disease Questionnaire.

As shown in Table 2, over the 1-year period of prospective observation, there were no major changes in PD assessment scores: Hoehn and Yahr PD stage, MDS-UPDRS Part III total score of motor examination, and NMSS score (non-motor symptomatology) remained at values indicative of moderate disease from T0 to T2. The mean PDQ-39 score showed a numerical improvement from 35 at T0 to 24 at T2.

**Table 2.** Changes in disease assessment scores during the 1-year follow-up.

	T0	T1	T2
Hoehn and Yahr stage	2.7 (0.8)	2.6 (0.8)	2.8 (0.8)
Motor examination score^a^ MDS-UPDRS, Part III, total score	37.6 (16.2)	36.8 (17.0)	37.6 (17.5)
NMSS	59.1 (43.2)	–	57.7 (38.2)
PDQ-39	35.0 (14.7)	–	24.0 (18.7)

Data are presented as mean values (SD).

^a^ Score according to MDS-UPDRS, Part III (range 0–108) [17].

MDS-UPDRS: Unified Parkinson’s Disease Rating Scale by the Movement Disorder Society; NMSS: Non-Motor Symptom Scale; PDQ-39: Parkinson’s Disease Questionnaire; SD: standard deviation; T0: study entry; T1: approximately 6 months since study entry; T2: approximately 12 months since study entry.

At T2, the most prevalent non-motor symptom as assessed by the physician using MDS-UPDRS Part IA was depressed mood, with 48% of patients showing mild-to-moderate symptoms, followed by anxious mood and apathy (respectively, 43% and 29% of patients with mild-to-moderate symptoms; Supplementary Materials, Figure S1A). The non-motor experiences of daily living (MDS-UPDRS Part IB) of mild-to-moderate severity that were most frequently reported by patients included fatigue (61% of patients), constipation (55%), and sleep problems (46%; Supplementary Materials, Figure S1B). The most frequent patient-reported daily motor problems (MDS-UPDRS Part II) of mild-to-severe intensity concerned handwriting (65%), getting out of bed, a car, or a deep chair (62%), dressing (58%), walking and balance (56%), turning in bed (56%), and speech (52%; Supplementary Materials, Figure S1C).

Motor complications (dyskinesias, motor fluctuations, and OFF-state dystonia) were assessed in Part IV of the MDS-UPDRS. The full set of data at T2, including a subanalysis by sex, is shown in Table 3. Dyskinesia affected 70% of patients and appeared to be more frequent in women than in men (77% in women vs 64% in men); women spent 26% of their waking time with dyskinesia compared with 20% for men. The functional impact of dyskinesia was moderate-to-severe in 53% of women and 42% of men. More than 90% of the study population had motor fluctuations (OFF state) for <50% of their waking time and >25% of their waking time was spent in the OFF-state, with a considerable functional impact (56% slight-to-mild impact; 37% moderate-to-severe impact), with no differences between sexes. About 35% of patients were affected by painful OFF-state dystonia; overall, patients spent 12% of their waking time with OFF-state dystonia.

**Table 3.** Assessment of motor complications (MDS-UPDRS part IV) at study end (T2)^a^.

	Total (*N* = 227)	Males (*n* = 135)	Females (*n* = 92)
**A. Dyskinesias (exclusive of OFF-state dystonia)**			
Time spent with dyskinesias, n (%)			
Normal	69 (30.5)	48 (35.8)	21 (22.8)
Slight-to-mild	125 (55.3)	68 (50.7)	57 (62.0)
Moderate-to-severe	32 (14.2)	18 (13.4)	14 (15.2)
Missing	1	1	0
Mean waking time spent with dyskinesias, %	22.3	19.8	25.8
Functional impact of dyskinesias, n (%)			
Normal	85 (37.6)	59 (44.0)	26 (28.3)
Slight-to-mild	36 (15.9)	19 (14.2)	17 (18.5)
Moderate-to-severe	105 (46.5)	56 (41.8)	49 (53.3)
Missing	1	1	0
**B. Motor fluctuations**			
Time spent in the OFF state, n (%)			
Normal	5 (2.2)	4 (3.0)	1 (1.1)
Slight-to-mild	209 (92.1)	125 (92.6)	84 (91.3)
Moderate-to-severe	13 (5.7)	6 (4.4)	7 (7.6)
Mean waking hours spent in OFF state, %	23.9	24.5	23.0
Functional impact of fluctuations, n (%)			
Normal	19 (8)	7 (5.2)	5 (5.4)
Slight-to-mild	139 (56)	83 (61.5)	49 (53.3)
Moderate-to-severe	94 (37)	45 (33.3)	38 (41.3)
Complexity of motor fluctuations, n (%)			
Normal	12 (5.3)	5 (3.7)	3 (3.3)
Slight-to-mild	184 (81.1)	111 (82.2)	73 (79.3)
Moderate-to-severe	35 (15.4)	19 (14.1)	16 (17.4)
**C. OFF dystonia**			
Painful OFF-state dystonia, n (%)			
Normal	149 (65.6)	89 (65.9)	60 (65.2)
Slight-to-mild	64 (28.2)	38 (28.1)	26 (28.3)
Moderate-to-severe	14 (6.2)	8 (5.0)	6 (6.5)
Mean OFF hours spent with dystonia, %	12.1	11.4	13.1

^a^ This assessment includes only patients using oral levodopa therapy at study entry. Normal is defined by the absence of dyskinesias/motor fluctuations/OFF dystonia; Slight, ≤ 25% of waking time spent with dyskinesias/motor fluctuations/OFF dystonia; Mild, 26–50% of waking time spent with dyskinesias/motor fluctuations/OFF dystonia; Moderate, 51–75% of waking time spent with dyskinesias/motor fluctuations/OFF dystonia; Severe, > 75% of waking time spent with dyskinesias/motor fluctuations/OFF dystonia.

MDS-UPDRS: Unified Parkinson’s Disease Rating Scale by the Movement Disorder Society.

### Types of motor fluctuations over 3 years

**Figure 2.**
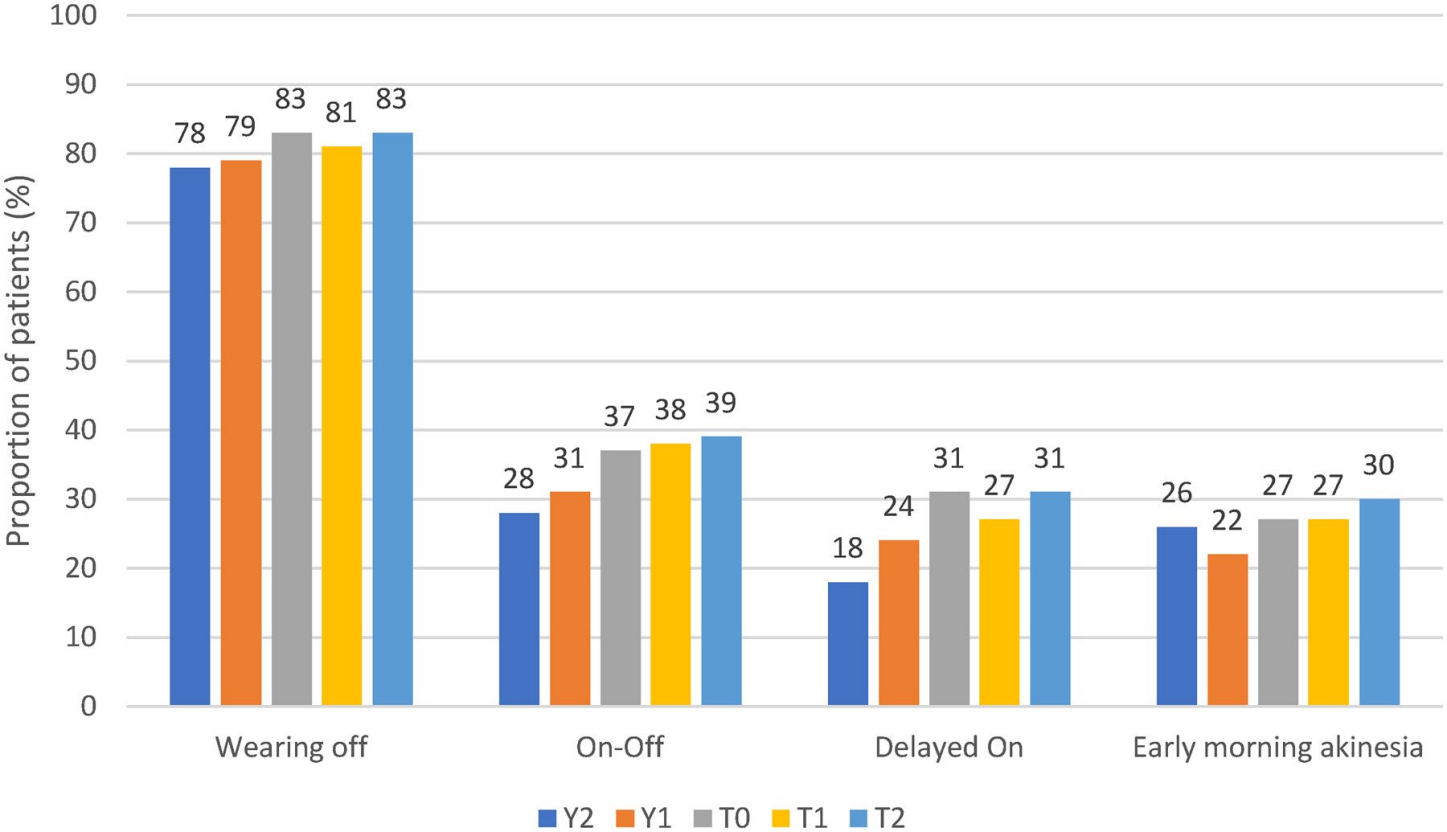
Types of motor fluctuations over the entire study period (3 years). T0: study entry; T1: approximately 6 months since study entry; T2: approximately 12 months since study entry; Y1: approximately 1 year before study entry; Y2: approximately 2 years before study entry.

Wearing-off of the response to levodopa was the most common type of fluctuation reported, with approximately 80% of the study population being affected over the 3-year study period (Figure 2). Other types of fluctuations, ON-OFF state (28–39%), delayed ON (18–31%), and early morning akinesia (26–30%) were less common. All motor fluctuation types showed a tendency to slightly increase in frequency over the observation period (Figure 2).

Motor and non-motor symptoms of wearing-off fluctuations were assessed with the WOQ-19 questionnaire (Supplementary Materials, Figure S2). The most common symptoms included slowness of movement (97% of patients), reduced dexterity (96%), general stiffness (85%), weakness (83%), difficulty in speech (71%), and problems with balance (70%). Most symptoms (12/19, 63%) improved after treatment with levodopa in >50% of patients. Some symptoms appeared to be more resistant to treatment, with ≤50% of patients reporting an improvement; these included problems with balance, mood changes, pain, cloudy mind, sweating, aching, and abdominal discomfort.

### Treatment over 3 years

At study entry, as per protocol, nearly all patients (99%) were in treatment with oral levodopa, with a mean daily dose of 662.2 mg and a mean number of daily administrations of 5.6 (Table 4). Only a minority of patients were using advanced therapies (5% of patients treated with infusion therapies; no patient treated with DBS).

**Table 4.** Characteristics of daily use of oral levodopa over the entire study period (3 years).

	Y2	T0	T2
Mean (SD) daily dose, mg	661.7 (308.4)	662.2 (271.5)	631.7 (291.5)
Mean (SD) daily number of administrations	–	5.6 (1.9)	–

SD: standard deviation; T0: study entry; T2: approximately 12 months since study entry; Y2: approximately 2 years before study entry.

**Figure 3.**
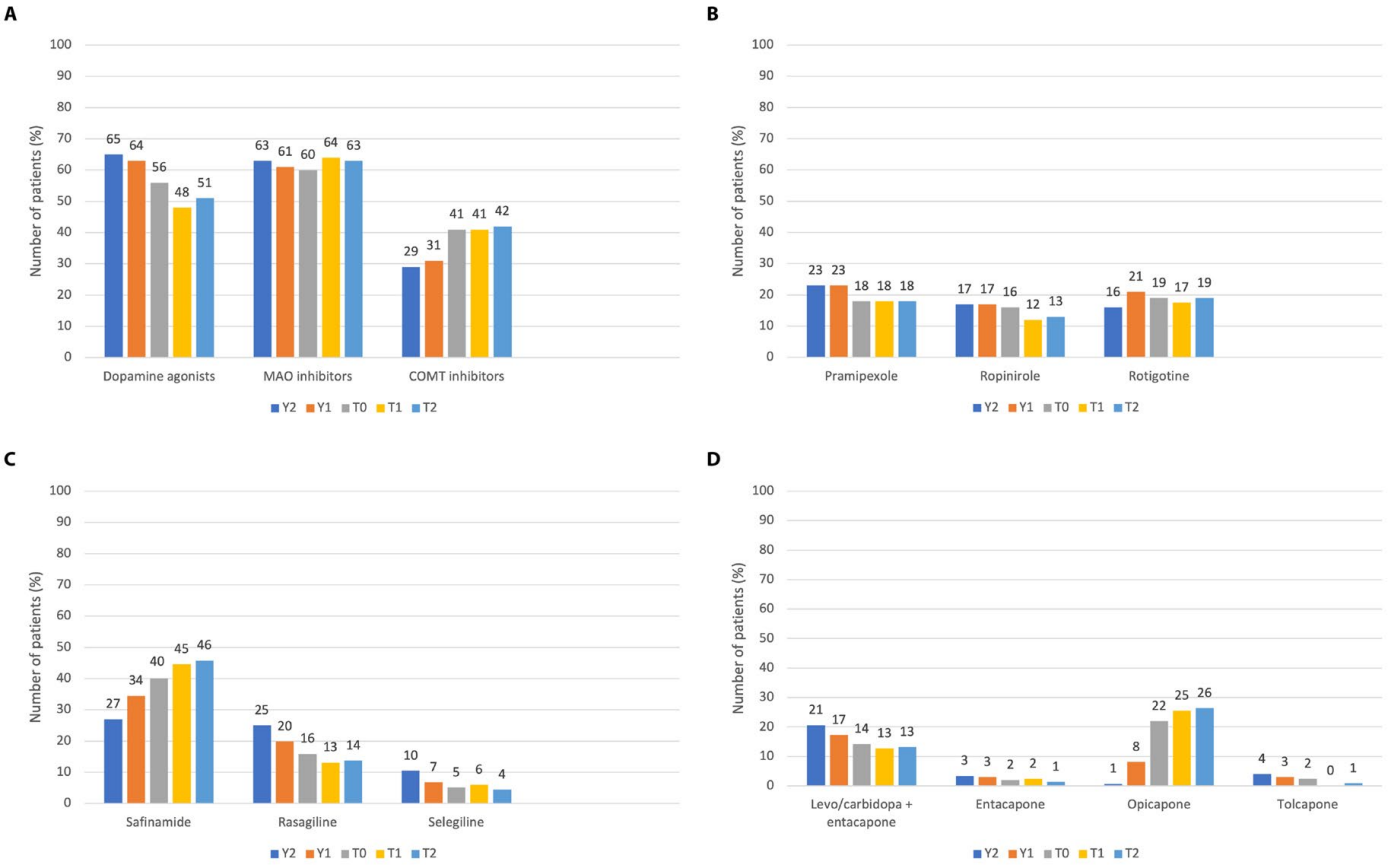
Add-on medications over the entire study period (3 years). **A.** Classes of add-on medications; **B.** Dopamine agonists; **C.** MAOB inhibitors; **D.** COMT inhibitors. COMT: catechol-O-methyltransferase; MAOB: monoamine oxidase-B; Levo: levodopa; T0: study entry; T1: approximately 6 months since study entry; T2: approximately 12 months since study entry; Y1: approximately 1 year before study entry; Y2: approximately 2 years before study entry.

The results of the analysis of the medications added to levodopa over the entire study period are summarized in Figure 3. MAOBi appeared to be the most frequently used class of add-on medications (used by 60% of the population at T0), followed by DA (56%) and COMTi (41%; Figure 3A). Over the 3-year period considered by the study, the use of DA appeared to be progressively decreasing (from 65% at Y2 to 51% at T2), while the use of MAOBi was stable (63% at Y2 and 63% at T2). The use of COMTi progressively increased from 29% at Y2 to 42% at T2. The use of the DA pramipexole, ropinirole, and rotigotine was quite balanced among the users of this class of add-on medications (Figure 3B). By contrast, data on the use of MAOBi showed that safinamide was largely preferred over rasagiline and selegiline (Figure 3C). Also, while the use of safinamide steadily increased over the 3-year study period (from 27% at Y2 to 46% at T2), the use of both rasagiline and selegiline showed a steadily decreasing trend (from 25% at Y2 to 14% for rasagiline and from 10% at Y2 to 4% at T2 for selegiline). As for COMTi, opicapone was the predominant drug used in the prospective phase of the study (22–26% of patients; Figure 3D). Over the 3-year study period, its use markedly increased from 1% at Y2 to 26% at T2; by contrast, the use of the combination levodopa/carbidopa plus entacapone progressively decreased from 21% at Y2 to 13% at T2.

**Figure 4.**
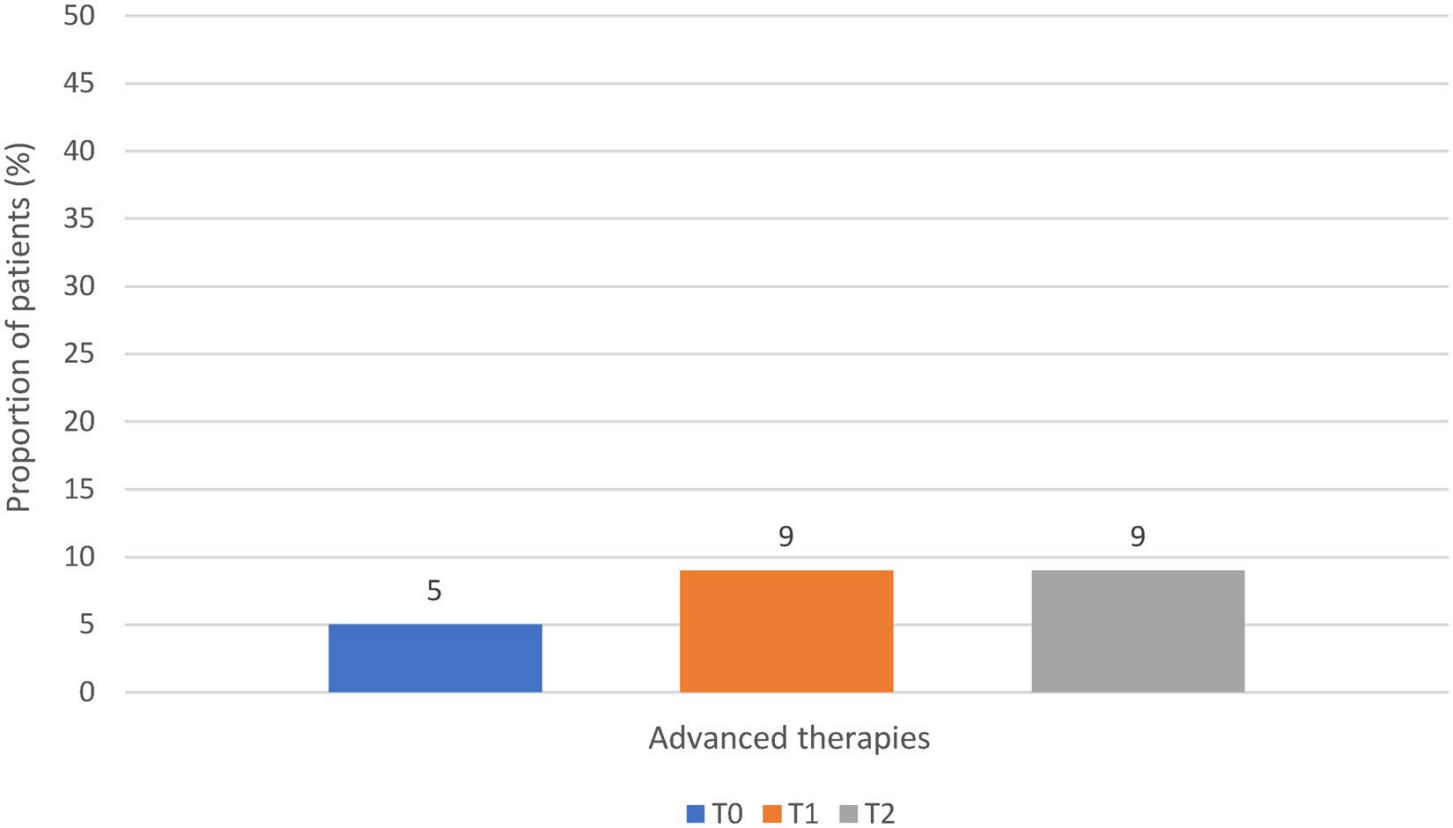
Use of advanced therapies (infusion therapies and deep brain stimulation). T0: study entry; T1: approximately 6 months since study entry; T2: approximately 12 months since study entry.

From T0 to T1 and T2, the proportion of patients using advanced PD therapies (infusion therapies and DBS) increased slightly but remained < 10% (Figure 4).

The proportions of patients needing a change in their therapy showed a decreasing trend over the 3-year study period (48% of patients at Y2, 54% at Y1, 51% at T0, 33% at T1, and 30% at T2; Supplementary Materials, Figure S3A). Fluctuations were the main reason for therapy change (>50% over the entire study period; Supplementary Materials, Figure S3B); of note, the number of patients changing therapy due to fluctuations tended to decrease over the 3-year study period (from 82% at Y2 to 61% at T2). The proportions of patients changing therapy due to tolerability problems ranged between 4% and 16% and the proportion of those changing therapy due to the need to control symptoms (mostly dyskinesias and hallucinations) ranged between 10% and 29% (Supplementary Materials, Figure S3B).

**Figure 5.**
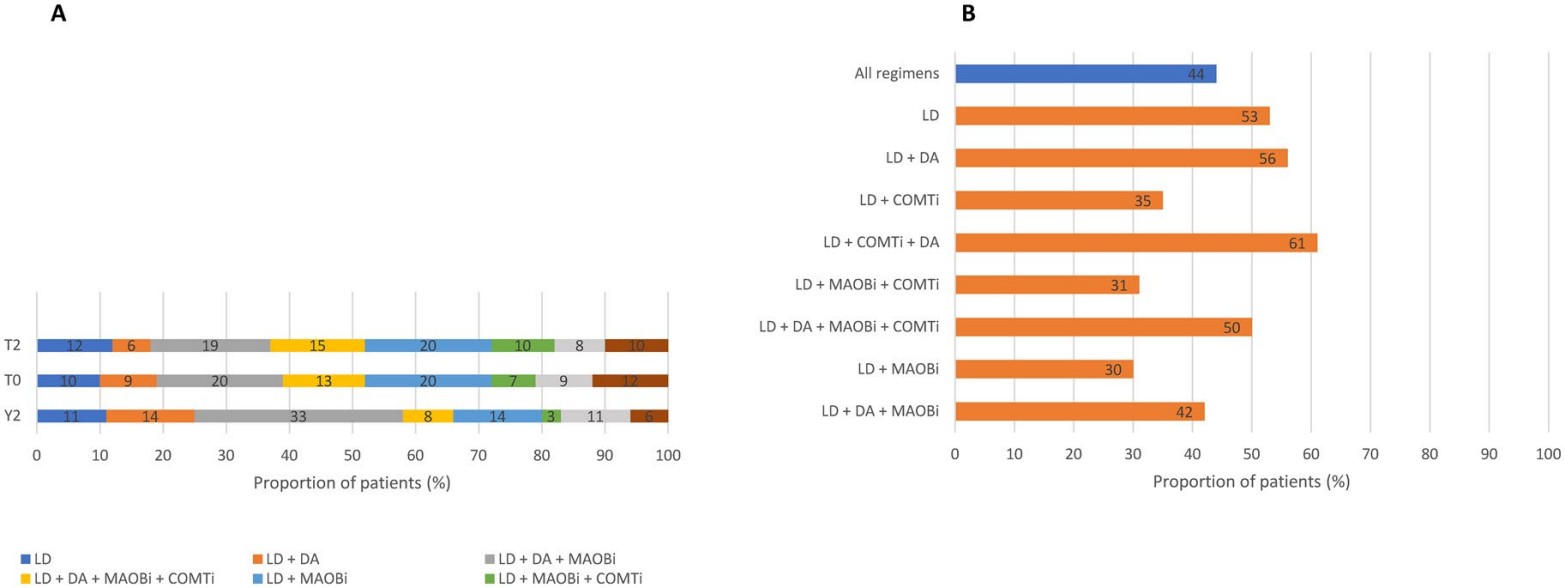
Analysis of treatment patterns in patients with advanced PD. In both analyses, patients receiving advanced therapy for PD were excluded. **A.** Use of therapeutic combinations over the 3-year observation period (Y2, *n* = 295 patients; T0, *n* = 292; T2, *n* = 222); **B.** Patients who needed a change of therapy. Patient percentages reported in panel B are the weighted mean of the values at the three time points. Ongoing oral levodopa-based combinations used in patients who were prescribed a new therapy at Y2 (*n* = 141), T0 (*n* = 148), and T2 (*n* = 66). COMTi: catechol-O-methyltransferase inhibitor; DA: dopamine agonist; LD: levodopa; MAOBi: monoamine oxidase-B inhibitor; T0: study entry; T1: approximately 6 months since study entry; T2: approximately 12 months since study entry; Y1: approximately 1 year before study entry; Y2: approximately 2 years before study entry.

The most common combinations across time points were levodopa- DA-MAOBi, followed by levodopa-MAOBi and levodopa-DA-MAOBi-COMTi. Only a minority of patients used levodopa monotherapy (10–12% of patients; Figure 5A). The combinations that were most commonly changed over the 3 years observational period were levodopa-COMTi-DA (61%), followed by levodopa-DA (56%) and levodopa monotherapy (53%), whereas the combinations that were associated with the lowest rates of treatment change were levodopa-MAOBi (30%), levodopa-MAOBi-COMTi (31%) and levodopa-COMTi (35%; Figure 5B).

## Discussion

We describe here a population of patients with long-standing PD (time since diagnosis >10 years), treated with levodopa and adjunctive PD medications, and experiencing response fluctuations for ≥2 years. The collected data encompassed a total of 3 years of treatment and follow-up at centers specialized in movement disorders across Italy.

Our patients had a mean age of 68 years, had been diagnosed at a mean age of 56 years, and almost half of them were affected by comorbidities, with a predominance of cardiovascular disease or risk factors, consistent with other reports describing PD populations [26]. All patients had motor fluctuations, with no substantial differences between male and female patients. Wearing-off was the predominant type of fluctuation. Dyskinesias affected approximately 70% of patients with a numerically greater frequency in women; the functional impact of dyskinesias appeared to be greater in women than in men. An increased risk of developing dyskinesia in women versus men has been reported in the literature [27], as well as in the interim analysis of the present study [16].

Overall, our patients had mild-to-moderate PD and were physically independent; their disease status, as assessed by different PD scoring systems was stable over the entire observation period. Of note, quality of life showed an improving trend from enrollment (PDQ-39 mean summary score, 35.0) to the end of observation (mean summary score, 24.0). Similar PDQ-39 summary scores have been reported in real-world studies in general PD populations. For example, in the PRISM European survey on the burden of PD (861 patients; mean age, 65 years; mean disease duration, 7.7 years), the median PDQ-39 score was 29.1 (interquartile range, 18.0–43.9) [28]. An earlier European study in 817 patients with PD (mean age, 66.5 years; mean disease duration, 3.3 years) reported a mean PDQ-39 score of 25.4 [8].

The results of the final analysis of our study confirm what emerged from the interim analysis of the retrospectively collected data [16]. Despite extended PD duration, older age, presence of relevant comorbidities, and frequent adjustments of PD medications (51% at T0, 33% at T1, and 30% at T2), patients did not show any sign of PD worsening/progression and their quality of life appeared to improve. On the other hand, substantial proportions of patients had their PD medications changed at each study visit (with fluctuations being the most frequent reason for change), indicating that there is a degree of complexity in off-period control. Furthermore, the assessment of motor and non-motor fluctuations using the WOQ-19 showed that for seven of the 19 symptoms considered (i.e. balance problems, mood changes, pain, cloudy mind, sweating, aching, and abdominal discomfort), <50% of patients had an improvement with levodopa treatment. Similar WOQ-19 results were reported in the interim analysis [16]. These data indicate that the progressive neurodegeneration in PD involves other systems than just dopaminergic pathways, and that some of the symptoms do not respond to levodopa.

With regard to the treatment used in patients with long-standing PD and fluctuations, only a minority of patients (<10%) used advanced therapies for PD including LCIG infusion, apomorphine infusion, and DBS; this is despite the fact that all the sites involved in the study were able to provide these therapies. Among add-on therapies, MAOBi were the most commonly prescribed class (63% of patients at the end of observation), followed by DA (51%) and COMTi (42%). While the use of MAOBi over the 3 years of observation was quite stable, the use of DA (they were the most frequently used add-on drugs at Y2 and Y1) progressively decreased and the use of COMTi progressively increased. Among MAOBi and COMTi, there was a clearly increasing trend in the use of safinamide and opicapone, respectively. Both drugs have been approved for the treatment of PD in the last 10 years, currently representing the latest generations of MAOBi and COMTi [3]. While the use of safinamide and opicapone is now well established for treating motor fluctuations [4,29–31], more research is needed to characterise their effects on non-motor fluctuations and before the onset of motor fluctuations [32]. In this regard, the EPSILON study showed that, over 24 weeks of treatment, opicapone reduced the severity of motor symptoms in levodopa-treated patients without motor complications [33]; data from the open-label extension phase are not yet available.

The present study also analysed the changes across 3 years of the combinations of PD medications, showing that the most commonly used combination was levodopa-DA-MAOBi. This trend is aligned with the results of PRISM study, where this triple combination was the commonest in Italy (18.7%) [26].

In PD, changing therapy frequently, for example 3–6 months after the introduction of a new treatment, can be considered as an indicator of suboptimal efficacy and/or poor tolerability of the new treatment strategy. In the present study, the analysis of the treatment patterns shows that the combinations of levodopa-MAOBi, levodopa-MAOBi-COMTi, and levodopa-COMTi led to fewer treatment changes. Notably, these combinations include the newest anti-PD medications, opicapone and safinamide, which may be more effective and better tolerated [34]. On the other hand, levodopa monotherapy and the combinations levodopa-DA, and levodopa-DA-MAOBi might be insufficient in patients with fluctuations. In these patients, the addition of COMTi has been shown to stabilize levodopa levels and to reduce fluctuations without increasing the risk of dyskinesias [31,35]. It appears that the use of drug combinations may be advantageous in providing longer durations of symptomatic control. In fact, such a strategy exploits the use of low doses of multiple medications rather than escalating levodopa doses [34]. However, with the quadruple combination levodopa-DA-MAOBi-COMTi, the dopaminergic activity might be excessive or the patients requiring it may have advanced disease resulting in tolerability problems, dyskinesias, and psychosis. The same might be true for the combination levodopa-COMTi-DA. Indeed, it is important when choosing an add-on therapy to evaluate not only the characteristics of the molecule in terms of efficacy and safety, but also the overall dopaminergic daily load, in terms of the levodopa equivalent dose (LED) [36]. An additional consideration with combination therapy is the possibility of drug-drug interactions [37], particularly if patients are receiving additional treatments for comorbidities or the relief of non-motor symptoms of PD such as autonomic dysfunction [38].

Several studies have addressed the patterns of treatment for PD in clinical practice [3,28,39,40]. A systematic review published in 2018 investigated the patterns and determinants of anti-PD drug prescriptions from 1967 to 2018 (44 studies, 17 countries) [3]. According to the systematic analysis, oral levodopa was the most common anti-PD medication prescribed during the entire period considered in all but four studies (37.4% to 100% of all anti-PD medications). DA were the second most commonly prescribed medications (prescribing rate from 7.6% to 85%) [3]. Prescriptions of MAOBi and COMTi varied substantially between countries (ranging from 2.1% to 42% and 1% to 29%, respectively) [3]. The international, observational study OBSERVE-PD was designed to describe the characteristics and outcomes of patients with advanced PD [39]. The analysis of the Italian subgroup of OBSERVE-PD compared treatment patterns of patients with advanced PD and non-advanced PD [28]. Patients with advanced PD were treated mostly with oral levodopa/carbidopa or benserazide (92%), followed by DA (58%), COMTi (37%), and MAOBi (23%); device-aided therapies (LCIG, DBS, apomorphine subcutaneous infusion) were used by 30% of patients [28]. The authors of that study noted the underuse of device-aided therapies in patients with advanced PD and persistent motor fluctuations.

The most common reason for changing treatment in our study was motor fluctuation, while other symptoms (including dyskinesias) were the second most common reason. It is notable that, at T2, the relative importance of motor fluctuations declined and that of other symptoms increased. This may indicate changes in symptomatology over time, even if overall PD scores show stable disease. The REASON study in Italy (2013) also reported motor symptom worsening as the leading cause of treatment modification in patients with PD (*n* = 775), but did not differentiate between motor fluctuations and other motor symptoms [41]. In contrast, research by Navaratnam and colleagues (2022) reported that wearing off/’on-off’ phenomena were the third most common reason for dose escalation or add-on therapy (after symptom relapse and perceived lack of efficacy) among Indian PD patients [42]. Drug-related adverse effects was the least common reason for treatment change in our analysis but was the most common reason for drug discontinuation or forward switch in the Indian study [42]. Discrepancies between the studies possibly arise at least in part from the different methodologies used to evaluate reasons for treatment change. Neither our study nor the Indian study assessed whether treatment change was impacted by considerations related to the patient’s current comorbidities or risk profile (e.g. the use of COMTi or decarboxylase inhibitors to limit changes in homocysteine levels among patients receiving levodopa [43,44]). This would be an interesting area of future research.

Therapeutic options in PD remain limited, and (as our study and others demonstrate) most physicians add multiple different treatments to levodopa over the course of the disease in response to the progressive degeneration of dopaminergic neurons in the substantia nigra and the evolving symptom profile [45]. Degeneration in dopaminergic pathways stimulates complex compensatory mechanisms within serotonergic, noradrenergic, and aminergic pathways [45], and dysfunction in midbrain serotonergic pathways is implicated in the development of levodopa-induced dyskinesias [46]. Further research into the role of these compensatory mechanisms has the potential to yield novel treatment approaches for PD, as does research into the role of neuroinflammation in PD development and progression. Until new treatments become available, research efforts are focused on enhancing the pharmacokinetic profiles of existing agents to optimize continuous drug delivery and tolerability. In addition to the development of new treatments or treatment delivery systems for PD, further research is needed in how best to tailor existing therapy for individual patients, based on their clinical profile and possibly genetic variants. The identification of biomarkers relevant to PD may be useful, not only for diagnosis and disease monitoring, but for optimizing treatment selection [47].

Our study has limitations. Being focused on clinical practice in Italy, the findings of our study may not apply to the management of PD patients in other countries. The study was performed in advanced PD centers therefore it may not reflect the general attitude of neurologists. The small number of patients receiving certain anti-PD medications did not allow efficacy comparisons between treatments. The significant loss of patients during follow-up (23%) may be attributable in part to the COVID-19 pandemic and related restrictions. Our analysis was limited to efficacy and QoL outcomes, and did not evaluate the impact of treatment on comorbidities or non-PD outcomes. Despite these limitations, we believe that our study provides useful information on current trends of real-world management of patients with long-standing PD, a subgroup that is poorly documented in the literature but frequently encountered in routine clinical practice.

Last but not least, the results of our study reinforce the importance of telemedicine, which provided an alternative tool to collect data at follow-up visits during the COVID pandemic. Despite a growing body of evidence supporting the effectiveness of telemedicine to manage PD patients, and the COVID pandemic that paved the way for its widespread adoption, the use of remote consultation is still limited in Italy [48,49].

## Conclusions

In long-standing PD, oral levodopa combined with adjunctive therapies remains the primary treatment for most patients. The results of the PD-FPA study suggest that oral medications can ensure the maintenance of an acceptable quality of life even in patients with advanced disease, provided patients are followed-up regularly and therapy adjustments are made as necessary. The availability of several effective add-on options, including new-generation MAOBi and COMTi can help in tailoring PD treatment to each patient, and the combination of multiple agents may be more advantageous than using monotherapy. However, when selecting treatment for advanced PD patients, it is important to consider not only the effectiveness and tolerability of treatments, but also factors such as severity of motor and non-motor symptoms, lifestyle, comorbidities and adherence to the entire therapeutic regimen, which in a patient at advanced stages of the disease may involve as many as 10 medications per day.

Until disease-modifying therapies become available for PD, symptomatic treatment remains the only available option. From this perspective, it would be important to conduct long-term studies evaluating whether better continuous dopaminergic stimulation through the early use of add-on therapy to levodopa could delay the onset, or reduce the severity, of motor complications.

## Supplementary Material

Stocchi_supplementary_19Dec24.docx

## Data Availability

The data supporting the findings of this study are available from the study sponsor (Bial Italy) or the Corresponding Author upon reasonable request (fabrizio.stocchi@sanraffaele.it; info.it@bial.com).

## References

[1] National Institute for Health and Care Excellence (NICE). Parkinson’s disease in adults (NG71). 2017. Available from: https://www.nice.org.uk/guidance/ng71/resources/parkinsons-disease-in-adults-pdf-1837629189061. Accessed April 12 2024.

[2] Fox SH, Katzenschlager R, Lim SY, et al. International Parkinson and movement disorder society evidence-based medicine review: update on treatments for the motor symptoms of Parkinson’s disease. Mov Disord. 2018;33(8):1248–1266. doi: 10.1002/mds.27372. 29570866

[3] Orayj K, Lane E. Patterns and determinants of prescribing for Parkinson’s disease: a systematic literature review. Parkinsons Dis. 2019;2019:9237181–9237140. doi: 10.1155/2019/9237181. 31781365 PMC6875178

[4] Fackrell R, Carroll CB, Grosset DG, et al. Noninvasive options for ‘wearing-off’ in Parkinson’s disease: a clinical consensus from a panel of UK Parkinson’s disease specialists. Neurodegener Dis Manag. 2018;8(5):349–360. doi: 10.2217/nmt-2018-0020. 29975112

[5] van der Velden RMJ, Broen MPG, Kuijf ML, et al. Frequency of mood and anxiety fluctuations in Parkinson’s disease patients with motor fluctuations: a systematic review. Mov Disord. 2018;33(10):1521–1527. doi: 10.1002/mds.27465. 30225905

[6] Armstrong MJ, Okun MS. Diagnosis and treatment of Parkinson disease: a review. JAMA. 2020;323(6):548–560. doi: 10.1001/jama.2019.22360. 32044947

[7] Stocchi F, Antonini A, Barone P, et al. Early DEtection of wEaring off in Parkinson disease: the DEEP study. Parkinsonism Relat Disord. 2014;20(2):204–211. doi: 10.1016/j.parkreldis.2013.10.027. 24275586

[8] Hechtner MC, Vogt T, Zöllner Y, et al. Quality of life in Parkinson’s disease patients with motor fluctuations and dyskinesias in five European countries. Parkinsonism Relat Disord. 2014;20(9):969–974. doi: 10.1016/j.parkreldis.2014.06.001. 24953743

[9] Rastgardani T, Armstrong MJ, Gagliardi AR, et al. Understanding, impact, and communication of “off” periods in Parkinson’s disease: a scoping review. Mov Disord Clin Pract. 2018;5(5):461–470. doi: 10.1002/mdc3.12672. 30515435 PMC6207105

[10] Santos‐García D, de Deus T, Cores C, et al. Levodopa-induced dyskinesias are frequent and impact quality of life in Parkinson’s disease: a 5-year follow-up study. Movement Disord Clin Pract. 2024;11(7):830–849. doi: 10.1002/mdc3.14056. PMC11233927 38747234

[11] Fabbri M, Barbosa R, Rascol O. Off-time treatment options for Parkinson’s disease. Neurol Ther. 2023;12(2):391–424. doi: 10.1007/s40120-022-00435-8. 36633762 PMC10043092

[12] Deuschl G, Antonini A, Costa J, et al. European academy of neurology/movement disorder society - European section guideline on the treatment of Parkinson’s disease: i. invasive therapies. Eur J Neurol. 2022;29(9):2580–2595. doi: 10.1111/ene.15386. 35791766

[13] Hely MA, Morris JG, Reid WG, et al. Sydney multicenter study of Parkinson’s disease: non-L-dopa-responsive problems dominate at 15 years. Mov Disord. 2005;20(2):190–199. doi: 10.1002/mds.20324. 15551331

[14] Yust-Katz S, Sthneer S, Melamed E, et al. Long-term Parkinson’s disease–time for optimism. Biomed Pharmacother. 2008;62(4):233–235. doi: 10.1016/j.biopha.2007.12.014. 18294809

[15] Garcia-Ruiz PJ, del Val J, Herranz A. Very long-standing Parkinson’s disease. Eur Neurol. 2013;70(3-4):172–174. doi: 10.1159/000351774. 23988401

[16] Stocchi F, Barone P, Ceravolo R, et al. Advanced Parkinson’s disease treatment patterns in Italy: an observational study interim analysis. Ann Med. 2024;56(1):2315226. doi: 10.1080/07853890.2024.2315226. 38381654 PMC10883087

[17] Hoehn MM, Yahr MD. Parkinsonism: onset, progression and mortality. Neurology. 1967;17(5):427–442. doi: 10.1212/wnl.17.5.427. 6067254

[18] Goetz CG, Tilley BC, Shaftman SR, et al. Movement disorder society-sponsored revision of the unified Parkinson’s disease rating scale (MDS-UPDRS): scale presentation and clinimetric testing results. Mov Disord. 2008;23(15):2129–2170. doi: 10.1002/mds.22340. 19025984

[19] Antonini A, Martinez-Martin P, Chaudhuri RK, et al. Wearing-off scales in Parkinson’s disease: critique and recommendations. Mov Disord. 2011;26(12):2169–2175. doi: 10.1002/mds.23875. 21780180

[20] Abbruzzese G, Antonini A, Barone P, et al. Linguistic, psychometric validation and diagnostic ability assessment of an Italian version of a 19-item wearing-off questionnaire for wearing-off detection in Parkinson’s disease. Neurol Sci. 2012;33(6):1319–1327. doi: 10.1007/s10072-012-0943-y. 22307444

[21] Chaudhuri KR, Martinez-Martin P, Brown RG, et al. The metric properties of a novel non-motor symptoms scale for Parkinson’s disease: results from an international pilot study. Mov Disord. 2007;22(13):1901–1911. doi: 10.1002/mds.21596. 17674410

[22] Cova I, Di Battista ME, Vanacore N, et al. Validation of the Italian version of the non motor symptoms scale for Parkinson’s disease. Parkinsonism Relat Disord. 2017;34:38–42. doi: 10.1016/j.parkreldis.2016.10.020. 28029554

[23] Peto V, Jenkinson C, Fitzpatrick R, et al. The development and validation of a short measure of functioning and well being for individuals with Parkinson’s disease. Qual Life Res. 1995;4(3):241–248. doi: 10.1007/BF02260863. 7613534

[24] Jenkinson C, Fitzpatrick R, Peto V, et al. The Parkinson’s disease questionnaire (PDQ-39): development and validation of a Parkinson’s disease summary index score. Age Ageing. 1997;26(5):353–357. doi: 10.1093/ageing/26.5.353. 9351479

[25] Galeoto G, Colalelli F, Massai P, et al. Quality of life in Parkinson’s disease: italian validation of the Parkinson’s disease questionnaire (PDQ-39-IT). Neurol Sci. 2018;39(11):1903–1909. doi: 10.1007/s10072-018-3524-x. 30088166

[26] Tolosa E, Ebersbach G, Ferreira JJ, et al. The Parkinson’s real-world impact assessment (PRISM) Study: a European survey of the burden of Parkinson’s disease in patients and their carers. J Parkinsons Dis. 2021;11(3):1309–1323. doi: 10.3233/JPD-212611. 34024784 PMC8461669

[27] Freitas ME, Hess CW, Fox SH. Motor complications of dopaminergic medications in Parkinson’s disease. Semin Neurol. 2017;37(2):147–157. doi: 10.1055/s-0037-1602423. 28511255 PMC5990008

[28] Stefani A, Tessitore A, Tambasco N, et al. Criteria for identification of advanced Parkinson’s disease: the results of the Italian subgroup of OBSERVE-PD observational study. BMC Neurol. 2022;22(1):41. doi: 10.1186/s12883-022-02554-z. 35090406 PMC8796340

[29] Valldeoriola F, Grandas F, Arbelo JM, et al. Spanish expert consensus on the use of safinamide in Parkinson’s disease. Neurologia (Engl Ed). 2018;36(9):666–672. doi: 10.1016/j.nrl.2018.04.007. 34752344

[30] Pagonabarraga J, Arbelo JM, Grandas F, et al. A Spanish consensus on the use of safinamide for Parkinson’s disease in clinical practice. Brain Sci. 2020;10(3):176. doi: 10.3390/brainsci10030176. 32197462 PMC7139287

[31] Antonini A, Barone P, Calabresi P, et al. The role of opicapone in the management of Parkinson’s disease: an Italian consensus through a combined nominal group technique and delphi approach. Eur Rev Med Pharmacol Sci. 2023;27(18):8850–8859. doi: 10.26355/eurrev_202309_33805. 37782207

[32] Regensburger M, Ip CW, Kohl Z, et al. Clinical benefit of MAO-B and COMT inhibition in Parkinson’s disease: practical considerations. J Neural Transm (Vienna). 2023;130(6):847–861. doi: 10.1007/s00702-023-02623-8. 36964457 PMC10199833

[33] Ferreira JJ, Rascol O, Stocchi F, et al. Opicapone as adjunct to levodopa in treated Parkinson’s disease without motor complications: a randomized clinical trial. Eur J Neurol. 2025;32(1):e16420. doi: 10.1111/ene.16420. 39790009 PMC11718218

[34] Stocchi F, Bravi D, Emmi A, et al. Parkinson disease therapy: current strategies and future research priorities. Nat Rev Neurol. 2024;20(12):695–707. doi: 10.1038/s41582-024-01034-x. 39496848

[35] Ferreira JJ, Poewe W, Rascol O, et al. Effect of opicapone on levodopa pharmacokinetics in patients with fluctuating Parkinson’s disease. Mov Disord. 2022;37(11):2272–2283. doi: 10.1002/mds.29193. 36054562 PMC9804871

[36] Jost ST, Kaldenbach MA, Antonini A, et al. Levodopa dose equivalency in parkinson’s disease: updated systematic review and proposals. Mov Disord. 2023;38(7):1236–1252. doi: 10.1002/mds.29410. 37147135

[37] Jost WH, Brück C. Drug interactions in the treatment of Parkinson’s disease. J Neurol. 2002;249 Suppl 3(0):III/24–29. doi: 10.1007/s00415-002-1305-0. 12522568

[38] Mallet L, Spinewine A, Huang A. The challenge of managing drug interactions in elderly people. Lancet. 2007;370(9582):185–191. doi: 10.1016/S0140-6736(07)61092-7. 17630042

[39] Fasano A, Fung VSC, Lopiano L, et al. Characterizing advanced Parkinson’s disease: OBSERVE-PD observational study results of 2615 patients. BMC Neurol. 2019;19(1):50. doi: 10.1186/s12883-019-1276-8. 30940119 PMC6444751

[40] Kasprzak J, Dulski J, Przytuła F, et al. Levodopa and dopamine agonist phobia in Parkinson’s disease - does it really matter? A survey on treatment patterns in Polish tertiary centres. Neurol Neurochir Pol. 2025;59(1):62–69. doi: 10.5603/pjnns.103168. 40007330

[41] Tinazzi M, Abbruzzese G, Antonini A, et al. Reasons driving treatment modification in Parkinson’s disease: results from the cross-sectional phase of the REASON study. Parkinsonism Relat Disord. 2013;19(12):1130–1135. doi: 10.1016/j.parkreldis.2013.08.006. 23993249

[42] Navaratnam P, Arcona S, Friedman HS, et al. Natural history and patterns of treatment change in Parkinson’s disease: a retrospective chart review. Clin Park Relat Disord. 2022;6:100125. doi: 10.1016/j.prdoa.2021.100125. 34950865 PMC8671728

[43] Müller T, Riederer P. The vicious circle between homocysteine, methyl group-donating vitamins and chronic levodopa intake in Parkinson’s disease. J Neural Transm. 2024;131(6):631–638. doi: 10.1007/s00702-023-02666-x. 37329350

[44] Müller T, Muhlack S. Peripheral COMT inhibition prevents levodopa associated homocysteine increase. J Neural Transm. 2009;116(10):1253–1256. doi: 10.1007/s00702-009-0275-0. 19657587

[45] Riederer P, Strobel S, Nagatsu T, et al. Levodopa treatment: impacts and mechanisms throughout Parkinson’s disease progression. J Neural Transm (Vienna). 2025;132(6):743–779. doi: 10.1007/s00702-025-02893-4. 40214767 PMC12116664

[46] Jeong EH, Lee JY, Song YS. Longitudinal serotonergic and dopaminergic binding: impact on Parkinson’s disease progression and levodopa dyskinesia. J Neuroimaging. 2025;35(1):e70014. doi: 10.1111/jon.70014. 39800858

[47] Yamashita KY, Bhoopatiraju S, Silverglate BD, et al. Biomarkers in Parkinson’s disease: a state of the art review. Biomark Neuropsychiatry. 2023;9:100074. doi: 10.1016/j/bionps.2023.100074.

[48] Ruggiero F, Lombi L, Molisso MT, et al. The impact of telemedicine on Parkinson’s care during the COVID-19 pandemic: an Italian online survey. Healthcare (Basel). 2022;10(6):1065. doi: 10.3390/healthcare10061065. 35742116 PMC9222237

[49] Cubo E, Delgado-López PD. Telemedicine in the management of Parkinson’s disease: achievements, challenges, and future perspectives. Brain Sci. 2022;12(12):1735. doi: 10.3390/brainsci12121735. 36552194 PMC9775481

