# Supplementary material for: Advanced Parkinson’s disease treatment patterns in Italy: results from a multicenter observational study
Source: Ann Med. 2026 Mar 4;58(1):2628353. doi: 10.1080/07853890.2026.2628353 (PMC12964474; doi:10.1080/07853890.2026.2628353)
Supplement: Stocchi_supplementary_19Dec24.docx [file IANN_A_2628353_SM6515.docx]

**Advanced Parkinson’s disease treatment patterns in Italy: results from a multicenter observational study**

**Supplementary Material**

**Authors:** Fabrizio Stocchi^1^, Paolo Barone^2^, Roberto Ceravolo^3^, Maria Francesca De Pandis^4-5^, Leonardo Lopiano^6^, Nicola Modugno^7^, Alessandro Padovani^8^, Manuela Pilleri^9^, Alessandro Tessitore^10^ and Mario Zappia^11^

**Affiliations:** ^1^Department of Neurology University San Raffaele Roma and IRCCS San Raffaele, 00166 Rome, Italy; ^2^University of Salerno, 84081 Baronissi, Salerno, Italy; ^3^Neurodegenerative Disease Center, Department of Clinical and Experimental Medicine, University of Pisa, 56126 Pisa, Italy; ^4^Department of Human Sciences and Promotion of Quality of Life, San Raffaele University, 00166 Roma, Italy; ^5^San Raffaele Cassino Hospital, 03043 Cassino, Italy; ^6^Department of Neuroscience Rita Levi-Montalcini, University of Turin; AOU Città della Salute e della Scienza, 10126 Turin, Italy; ^7^I.R.C.C.S. Neuromed, 86077 Pozzilli, Isernia, Italy; ^8^University of Brescia, 25100 Brescia, Italy; ^9^UO Neurologia Casa di Cura Villa Margherita, 36057 Arcugnano Vicenza, Italy and Centro Parkinson e Parkinsonismi ASST Gaetano Pini CTO, 20122 Milano, Italy; ^10^Department of Advanced Medical and Surgical Sciences, University of Campania "L. Vanvitelli", 80138 Naples, Italy; ^11^Dept “G.F. Ingrassia”, University of Catania, 95123 Catania, Italy

**Corresponding author:** Fabrizio Stocchi, Department of Neurology, University San Raffaele Roma and IRCCS San Raffaele, via della Pisana 235, 00163 Rome, Italy. Tel: +390652252311; Email: [fabrizio.stocchi@sanraffaele.it](mailto:fabrizio.stocchi@sanraffaele.it)

## **Supplementary Table S1.**

Overview of data collection

|  | **Retrospective phase** | | **Enrollment** | **Prospective phase** | |
| --- | --- | --- | --- | --- | --- |
|  | **Y2** | **Y1** | **T0** | **T1** | **T2** |
| Number of patients | 296 | 296 | 296 | 252 | 227 |
| Demographic data |  |  | x |  |  |
| Age at diagnosis |  |  | x |  |  |
| Age at fluctuation onset |  |  | x |  |  |
| Fluctuation type | x | x | x | x | x |
| Comorbidities |  |  | x | x | x |
| WOQ-19 |  |  | x | x | x |
| nM-EDL (MDS-UPDRS Part I) |  |  | x | x | x |
| M-EDL (MDS-UPDRS Part II) |  |  | x | x | x |
| MDS-UPDRS Part III |  |  | x | x | x |
| Hoehn and Yahr |  |  | x | x | x |
| MDS-UPDRS Part IV |  |  | x | x | x |
| NMSS |  |  | x |  | x |
| PDQ-39 |  |  | x |  | x |
| Ongoing pharmacologic therapy (total levodopa dose and name of active principles) at presentation | x | x | x | x | x |
| Change of pharmacologic therapy at the end of visit (Yes/No and reasons) | x | x | x | x | x |
| Interventions (DBS) |  |  | x^a^ | x | x |

^a^Patients with ongoing or previous DBS treatment at T0 were not enrolled.

DBS: deep brain stimulation; M-EDL: motor experiences of daily living; MDS-UPDRS: Unified Parkinson’s Disease Rating Scale revised by the Movement Disorder Society; nM-EDL: non-motor experiences of daily living; NMSS: Non-Motor Symptom Scale ; PDQ-39: Parkinson’s Disease Questionnaire; T0: study entry; T1: approximately 6 months since study entry; T2: approximately 12 months since study entry; WOQ-19: 19-item Wearing-Off Questionnaire; Y1: approximately 1 year before study entry; Y2: approximately 2 years before study entry.

## **Supplementary Figure S1.**

Non-motor and motor symptoms of Parkinson’s disease as assessed by MDS-UPDRS Parts I and II at T2

**A.** MDS-UPDRS Part IA (physician assessed), non-motor experiences of daily living (nM-EDL); **B.** MDS-UPDRS Part IB (patient assessed), nM-EDL; **C.** MDS-UPDRS Part II (patient assessed), motor experiences of daily living (M-EDL).

**A.**

**B.**

**C.**

## **Supplementary Figure S2.**

Motor and non-motor symptoms of wearing-off fluctuations as assessed by the 19-item Wearing-Off Questionnaire (WOQ-19) and their improvement after treatment at T2

## **Supplementary Figure S3.**

**A.** Changes of therapy over the entire study period; **B.** reasons for the change in therapy

^a^Symptoms included dyskinesias, muscle pain, psychiatric disorders (hallucinations, impulse control disorders, psychosis, disperception, depression), worsening or poor control of motor symptoms (stiffness, bradykinesia, freezing, nocturnal or morning akinesia).

T0: study entry; T1: approximately 6 months since study entry; T2: approximately 12 months since study entry; Y1: approximately 1 year before study entry; Y2: approximately 2 years before study entry.

**A.**

**B.**
